# Robotic Transhiatal Revision of Gastric Conduit After Esophagectomy: Experience in 15 Patients

**DOI:** 10.1016/j.atssr.2025.11.004

**Published:** 2025-11-25

**Authors:** SangMin Kim, James D. Luketich, Edwin Gutierrez, Inanc Samil Sarici, Yota Suzuki, Christian A. Otero, Matthew J. Schuchert, Ryan M. Levy, Omar Awais, Evan T. Alicuben

**Affiliations:** Division of Thoracic and Foregut Surgery, Department of Cardiothoracic Surgery, University of Pittsburgh Medical Center, Pittsburgh, Pennsylvania

## Abstract

**Background:**

The literature offers limited guidance on revision strategies for gastric conduit dysfunction after esophagectomy, traditionally approached through open or combined thoracoscopic-laparoscopic methods. We present our institution's early experience with robot-assisted transhiatal revisional surgery for severe conduit dysfunction.

**Methods:**

A retrospective, single-institution review was conducted of patients who underwent robot-assisted transhiatal revision of the gastric conduit after esophagectomy between January 1999 and April 2024.

**Results:**

We identified 15 patients (mean age, 64.1 years). All had redundant conduit anatomy, with or without angulation or a supradiaphragmatic shelf; dysphagia was the primary symptom. Paraconduit hernia was present in 6 patients (40%). The median time from the index esophagectomy to revision was 62 months. Median hospital stay was 3 days. Two patients (13.3%) experienced perioperative complications: 1 port-site hernia and 1 pneumonia and pulmonary embolism requiring reintubation and anticoagulation. One patient required redo revision for reherniation due to vigorous coughing. One patient died of sudden death at home after an unremarkable 4-day postoperative course. At a median follow-up of 5.3 months, the 14 surviving patients reported at least partial symptom resolution and were no longer dependent on supplemental feeding.

**Conclusions:**

Robot-assisted transhiatal revision of gastric conduit dysfunction after esophagectomy is feasible and safe, offering shorter hospital stays and acceptable perioperative morbidity. Although complete symptom resolution remains difficult to achieve, most patients experienced meaningful improvement in swallowing and nutritional independence. Further studies are warranted to improve outcomes in this complex patient population.

**In Short**

▪ Robotic transhiatal revision of redundant gastric conduits is technically feasible and can provide meaningful symptom relief in carefully selected patients.
▪ Enhanced visualization and precise mediastinal dissection with the robotic platform may allow safe reconstruction without thoracic access.

Emerging evidence indicates that surgical revision of the gastric conduit may be associated with acceptable perioperative morbidity and favorable functional outcomes.^1^, ^2^, ^3^, ^4^, ^5^, ^6^, ^7^, ^8^ Our institution previously reported a combined thoracoscopic and laparoscopic approach for gastric conduit revision in 43 patients with gastric conduit dysfunction, achieving symptomatic improvement in 85% of patients with dilated conduits and durable hernia repair in 71% of patients with type IV paraconduit hernias at a median follow-up of 12 months.^4^

Building on this experience, we hypothesized that a fully laparoscopic, robot-assisted approach could further minimize operative morbidity while preserving comparable functional outcomes. This study presents our institution’s initial experience with this technique in a cohort of 15 patients who underwent robot-assisted transhiatal revisional surgery.

## Material and Methods

We performed a single-institution, retrospective review of all patients who underwent robot-assisted transhiatal revision of gastric conduit after esophagectomy between January 1999 and April 2024. Patients with nongastric conduit, history of bipolar exclusion with cervical esophagostomy, previous conduit revision, or active malignancy were excluded. This study was approved with waiver of consent by the University of Pittsburgh Medical Center Institutional Review Board (IRB #: STUDY20050010).

### Diagnostic Workup and Nonoperative Management

When patients present with severe symptoms indicative of gastric conduit dysfunction after esophagectomy, our initial diagnostic approach centers on a barium swallow study. After the barium swallow esophagram, we typically proceed with computed tomographic imaging. For esophageal cancer survivors, this step serves a dual purpose: it allows us to rule out local and distant cancer recurrence while simultaneously providing valuable information for surgical planning in what could be a complex reoperative procedure.

Esophagogastroduodenoscopy (EGD) plays a pivotal role in our diagnostic and therapeutic approach to conduit revisional surgery. From a diagnostic standpoint, this procedure serves multiple purposes. First, it helps exclude intraluminal causes of delayed gastric emptying, such as stenosis, anastomotic stricture, tumor recurrence, or ulceration. Second, it provides direct visual evidence of significant delayed emptying (eg, retained food particles or fluid). Third, it allows further investigation into the etiology of delayed emptying, including conduit twisting, distal shelving at the hiatus, or a sigmoidal/tortuous conduit configuration that may be more amenable to surgical correction and predicts successful outcome after revisional surgery. As a standard practice during all endoscopies, we perform dilation under guidewire guidance. A favorable response to dilation typically obviates the need for surgical intervention.

## Robotic Transhiatal Gastric Conduit Revision: Operative Steps

**Figure 1:**
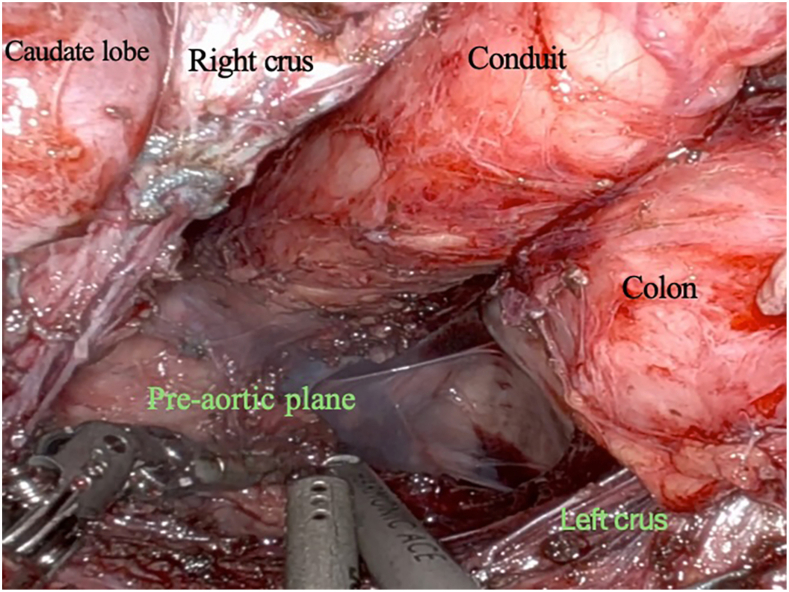
Mediastinal dissection through transhiatal surgical access using an ultrasonic energy device.

**Figure 2:**
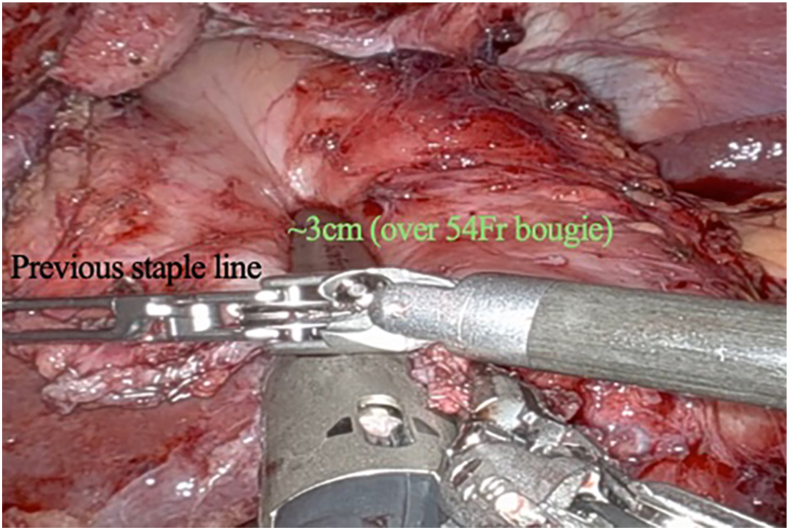
Robotic stapling of the conduit using a 54F bougie ensuring a consistent ∼3-cm width.

The robotic transhiatal conduit revision, performed entirely through laparoscopic incisions, includes adhesiolysis, diaphragmatic and hiatal mobilization, high mediastinal dissection, hernia reduction, conduit restapling, and hiatal closure with conduit pexy (Figure 1). The goal is to reconstruct a narrow, straight gastric conduit positioned within the mediastinum, perform cruroplasty and restore the phrenoesophageal ligament with near-circumferential pexy. Endoscopy confirms preoperative findings and guides mobilization. For conduit restapling, a 54F Maloney bougie is first passed down the esophagus; then, using the robotic stapler, we follow the curvature of the bougie to staple along the lesser curvature, proceeding cephalad to the extent of the conduit redundancy (Figure 2).

## Results

### Patient Characteristics and Index Esophagectomy

Fifteen patients underwent robot-assisted transhiatal revisional surgery for severe gastric conduit dysfunction (Table 1). Mean patient age was 64.1 years, with 11 (77%) having significant comorbidity according to the Charlson Comorbidity Index classification. Two-thirds had undergone esophagectomy for benign disease (primarily after failed antireflux surgery) and one-third for malignancy. Most were minimally invasive Ivor Lewis procedures, almost all with pyloric intervention. Median time from the index esophagectomy to revision was 62 months. Two patients were referred after esophagectomy at another institution.

**Table 1:** Patient Demographic, Clinical, Pathologic, and Surgical Profiles

Characteristics	Data Values (N = 15)
Age, mean, y	64.1
Female sex	7 (47.1)
Indication for index esophagectomy	
Malignant	5 (33.3)
Benign	10 (66.7)
Hiatal hernia/reflux	8
Achalasia	2
Type of esophagectomy	
Ivor Lewis	13 (86.7)
McKeown	2 (13.3)
Approach	
Minimally invasive	14 (93.3)
Open	1 (6.7)
Pyloric intervention	14 (93.3)
Anastomosis type	
Stapled	14 (93.3)
Hand-sewn	1 (6.7)
Time from index to revision, median, mo	62
Anatomic variance before revision	
Paraconduit hernia	6 (40)
Redundant conduit ± angulation/shelf	15 (100)
Presenting symptoms for revision	
Dysphagia	15 (100)
Nausea/vomiting	7 (46.7)
Dyspepsia	5 (33.3)
Severe weight loss/failure to thrive	3 (20.0)
Regurgitation	2 (13.3)
Aspiration pneumonia	1 (6.7)
Dyspnea	1 (6.7)
Nutritional status	
Tube feeding required	3 (20)

Data are presented as n (%) unless indicated otherwise.

### Preoperative Presentation and Indications for Revision

All patients presented with dysphagia, often accompanied by nausea and/or reflux. One-fifth required supplemental feeding. Imaging and endoscopy demonstrated redundant conduit anatomy in all patients, with 6 (40%) harboring type IV paraconduit hernia, of which 4 (26.7%) presented with colon herniation, 1 (6.7%) with colon and small bowel herniation, and 1 (6.7%) with small bowel herniation.

### Perioperative Outcomes

Median hospital stay was 3 days. Complications developed in 2 patients (13%), comprising 1 port-site hernia and 1 pneumonia/pulmonary embolism requiring reintubation. One patient required redo revision for reherniation. One patient died of sudden death at home after an unremarkable discharge. One patient required 30-day readmission (Table 2).

**Table 2:** Perioperative Outcomes and Follow-Up

Characteristics	Data Value (N = 15)
Complications	2 (13.3)
Clavien-Dindo Grade I	0 (0)
Clavien-Dindo Grade II	1 (6.7)
Clavien-Dindo Grade III	1 (6.7)
Leak	0 (0)
Length of stay, median, d	3
30-day readmission	1 (6.7)
30-day mortality	1 (6.7)
Postoperative quality of life	(n = 14)
Follow-up, median, mo	5.3
Symptom resolution	
Complete	2 (14.3)
Partial	12 (85.7)
Oral tolerance at follow-up	14 (100)
Residual symptoms	
Dysphagia	7 (50)
Dyspepsia	4 (28.6)
Regurgitation	2 (14.2)
Nausea/vomiting	3 (21.4)
Shortness of breath	1 (7.1)
Epigastric/chest pain	3 (21.4)
Reinterventions	
Esophagogastroscopy with dilation	5 (35.7)
Redo conduit revision	1 (7.1)

Data are presented as n (%) unless indicated otherwise.

### Postoperative Quality of Life and Symptom Resolution

At a median follow-up of 5.3 months, all surviving patients tolerated oral intake without feeding support. Most reported partial but meaningful symptom relief, and 2 achieved complete resolution. Dysphagia and dyspepsia were the most common residual complaints, and one-third required repeat endoscopic dilation (Table 2).

### Comparative Analysis in Benign vs Malignant Subgroups

Patients revised after malignant esophagectomy were older (mean 73 vs 60 years) but required fewer postoperative interventions and had higher rates of complete symptom resolution. In the benign group, 50% (5 of 10) presented with type IV paraconduit hernia, and all had a redundant conduit. In the malignant group, all 5 presented redundant conduits with 20% (1 of 5) also having type IV herniation.

All 3 perioperative complications, including one redo conduit revision, occurred in the benign group. The malignant group experienced a single death after an otherwise uneventful 4-day hospitalization. Median hospital stay was 3 days in both groups.

All patients reported at least partial improvement. Complete symptom resolution occurred in 50% (2 of 4) of the malignant group vs in none of the benign group. At 5.3 months’ median follow-up, no malignant patients required postoperative dilation or reoperation, whereas 50% (5 of 10) of benign patients underwent EGD with dilation.

## Comment

In this case series of 15 patients undergoing robot-assisted transhiatal revision for severe gastric conduit dysfunction, we demonstrate that the robotic approach can be a safe and effective option for carefully selected patients.

We apply 3 criteria when considering conduit revision. First, we confirm that the patient's symptoms are refractory to nonoperative and endoscopic interventions, such as serial dilations. Second, we confirm functional and cardiopulmonary reserve. Most critically, we identify a clear anatomical target—conduit redundancy, a fixed shelf, or paraconduit hernia—as the culprit of symptoms amenable to surgical repair.

In our experience, redundant conduit anatomy is the most significant contributor to gastric conduit dysfunction and represents a reliable target for surgical revision. In a prior study of 21 patients with severe symptomatic redundancy, 85% achieved at least partial symptom relief after revision, findings consistent with our cohort in which all patients reported improvement.^4^ Early clinical experiences across several centers have also shown encouraging outcomes with surgical revision for redundant gastric conduits.^5^^,^^6^ In contrast, isolated paraconduit hernias may not always present with symptoms indicating gastric conduit dysfunction. Although paraconduit hernias carry a theoretical risk of strangulation or other complications, the literature suggests catastrophic events are uncommon with expectant management in asymptomatic patients.^1^^,^^3^ Accordingly, we believe operative repair is best reserved for symptomatic patients or carefully selected high-risk asymptomatic cases. Six patients (40%) in our series had concomitant type IV hernias along with dilated conduit, most commonly involving the colon.

Redundant gastric conduits are commonly associated with obstructive symptoms such as dysphagia, nausea, vomiting, and weight loss. In our series, dysphagia was universal, and nearly half had nausea/vomiting. This constellation, when paired with identifiable anatomic abnormalities, predicts favorable outcomes.

The robotic transhiatal revisional approach aims to restore normal foregut physiology, while minimizing morbidity. Using enhanced visualization and superior instrument articulation, the dilated conduit is mobilized into the abdomen and reconstructed into a narrower, straighter profile within the mediastinum, improving gastric emptying by reducing negative intrathoracic pressure and enhancing bolus propulsion. Cruroplasty restores part of the antireflux barrier, pyloroplasty relieves outlet obstruction, and conduit-hiatal pexy prevents recurrent herniation. Robotic dissection allows safe mobilization to the anastomosis, avoiding thoracotomy or thoracoscopy and reducing morbidity associated with high mediastinal access.

Not all patients with redundant conduits benefit equally from revision. In our series, those with prior benign esophagectomy had worse outcomes. None achieved complete symptom resolution, whereas half of the malignant group did. At 5.3 months of follow-up, no patients in the malignant group required dilation or redo surgery, whereas half of the benign group did. Although all patients reported partial improvement, benign esophagectomy carries higher morbidity, and long-term functional outcomes remain less well defined. These findings highlight the need for further studies to identify predictors of surgical success.

In our cohort, robotic transhiatal conduit revision yielded outcomes comparable to previously reported revisional techniques, with the added benefit of shorter hospital stays. The median length of stay was 3 days, which compares favorably to historical reports of 8 to 10 days with open or thoracoscopic revisions.^5^^,^^6^ Perioperative morbidity was low, limited to 13.3% (2 of 15), including 1 port-site hernia and 1 pneumonia with pulmonary embolism. Importantly, no leaks, necrosis, or conduit perforations occurred. At a median follow-up of 5.3 months, all patients tolerated oral intake and were free from supplemental nutritional support. These results support the feasibility of performing effective conduit revision using only laparoscopic incisions without compromising adherence to core surgical principles.

This study is limited by its retrospective nature, small sample size, and performance by only 2 surgeons, all of which constrain the generalizability. The absence of long-term quality of life data >5.3 months limits assessment of the durability of symptom relief. Future work should emphasize prospective data collection and comparisons with nonsurgical strategies, including endoscopic approaches, to better define the role of revisional surgery.

Severe gastric conduit dysfunction after esophagectomy remains a complex and heterogeneous problem. Our experience suggests that robot-assisted transhiatal revision is a safe, technically feasible option that can provide meaningful symptom improvement in selected patients. Ongoing refinement of technique, greater understanding of delayed conduit dysfunction, and rigorous prospective evaluation will be essential to optimizing outcomes in this challenging population.
